# Comparative Analysis of Extracorporeal Shockwave Therapy, Bisphosphonate, and Wharton Jelly-Derived Mesenchymal Stem Cells in Preserving Bone and Cartilage Integrity and Modulating IL31, IL33, and BMP2 in the Cartilage of Ovariectomized Rat Model

**DOI:** 10.3390/biomedicines12122823

**Published:** 2024-12-12

**Authors:** Jai-Hong Cheng, Cheng-Wei Chen, Wen-Yi Chou, Po-Cheng Chen, Kuan-Ting Wu, Shun-Wun Jhan, Shan-Ling Hsu, Yi-No Wu, Hou-Tsung Chen

**Affiliations:** 1 Center for Shockwave Medicine and Tissue Engineering, Kaohsiung Chang Gung Memorial Hospital, Chang Gung University College of Medicine, Kaohsiung 833, Taiwan; cjh1106@cgmh.org.tw (J.-H.C.); murraychou@yahoo.com.tw (W.-Y.C.); enemy7523@gmail.com (K.-T.W.); b9502077@cgmh.org.tw (S.-W.J.); hsishanlin@yahoo.com.tw (S.-L.H.); 2 Medical Research, Kaohsiung Chang Gung Memorial Hospital, Chang Gung University College of Medicine, Kaohsiung 833, Taiwan; 3 Department of Orthopedic Surgery, Sports Medicine, Kaohsiung Chang Gung Memorial Hospital, Chang Gung University College of Medicine, Kaohsiung 833, Taiwan; jwchen@cgmh.org.tw; 4 Department of Physical Medicine and Rehabilitation, Kaohsiung Chang Gung Memorial Hospital, Chang Gung University College of Medicine, Kaohsiung 833, Taiwan; b9302081@cgmh.org.tw; 5 School of Medicine, Fu Jen Catholic University, New Taipei City 242, Taiwan; 133838@mail.fju.edu.tw; 6 Department of Leisure and Sports Management, Cheng-Shiu University, Kaohsiung 833, Taiwan

**Keywords:** osteoporosis, ESWT, bisphosphonate, WJMSCs, inflammation

## Abstract

**Background:** Osteoporosis (OP) is a chronic inflammatory bone disease characterized by reduced bone structure and strength, leading to increased fracture risk. Effective therapies targeting both bone and cartilage are limited. This study compared the therapeutic effects of extracorporeal shockwave therapy (ESWT), bisphosphonate (Aclasta), and human Wharton jelly-derived mesenchymal stem cells (WJMSCs) in a rat model of OP. **Methods:** Female rats were assigned to four groups: Sham (no surgery or treatment), OP (bilateral ovariectomy, OVX), ESWT (OVX + ESWT on both tibias at 0.25 mJ/mm^2^, 1500 impulses per tibia), Aclasta (OVX + zoledronic acid 0.1 mg/kg via tail vein injection), and WJMSC (OVX + 2 × 10⁶ WJMSCs). Pathological changes, bone microarchitecture (by micro-CT), serum cytokines (by ELISA), and tissue-specific molecular markers (by immunohistochemistry) were evaluated. **Results:** All treatments improved bone density, preserved cartilage, and modulated cytokines (IL31, IL33, VEGF, and BMP2), with Aclasta showing the greatest improvements in bone parameters and cartilage preservation. ESWT and WJMSC also demonstrated significant effects, with ESWT highlighting non-invasive chondroprotective potential. **Conclusions:** Aclasta provided the best overall therapeutic response, particularly in bone regeneration. However, ESWT and WJMSC also showed comparable chondroprotective effects. ESWT emerges as a promising non-invasive alternative for OP management when pharmacological or cell-based therapies are not feasible.

## 1. Introduction

Osteoporosis (OP) is a chronic, bone metabolic disease that causes the bones of humans to become less dense and more brittle [1]. Osteoporosis lowers quality of life, especially in the elderly people and postmenopausal women, as it results in 8.9 million fractures worldwide [1,2]. Various factors, such as aging, estrogen deficiency, medications, smoking, inflammation, and heredity for osteoporosis, adversely impact bone health [3]. As a result, improving bone health is thought to be a crucial issue for those who are more susceptible to osteoporosis. To stop or reverse bone loss, many medical and pharmaceutical interventions have been developed [4,5]. For example, weight-bearing aerobics exercise, resistance training, aquatic exercise, and mechanical stress are the physical approaches to decrease the rate of bone loss and improve bone mineral density [4,6,7]. The physical methods need to include regular daily exercise or stimulation through the use of mechanical devices and important supplements including calcium, vitamin D, and dietary nutrients for bone recovery. Osteoporosis drugs are safe, consistently very successful, and work more swiftly to treat the condition. Current pharmacological treatments for osteoporosis include anti-resorptive (bisphosphonates and denosumab), bone-forming (PTH analogues and PTHrP analogues), and bone-sparing (Wnt antagonists) treatments [5,8,9]. The pathology of osteoporosis is caused by an imbalance in the cellular functions of bone resorption by osteoclasts and bone formation by osteoblasts, resulting in reduced bone mass and increased fracture risk [10]. Maintaining the balance of bone modeling and remodeling is critical, and this is achieved through the contributions of various cell types, including osteocytes, osteoclasts, and osteoblasts [11].

Bisphosphonates, such as zoledronic acid, are widely used as anti-resorptive agents in the management of OP [12]. They function by inhibiting osteoclast-mediated bone resorption, thereby reducing bone turnover and increasing BMD. Zoledronic acid has been demonstrated to significantly reduce the risk of vertebral, hip, and other fractures in patients with OP. In addition to its effects on bone metabolism, zoledronic acid may have a protective role in preserving joint cartilage by reducing inflammation and promoting subchondral bone health [13]. Despite these benefits, bisphosphonates primarily target bone resorption and may not fully address the regenerative aspects of bone and cartilage.

In contrast, human Wharton Jelly-derived mesenchymal stem cells (WJMSCs) offer a regenerative approach to treating OP due to their ability to differentiate into osteoblasts and chondrocytes, as well as their anti-inflammatory and immunomodulatory properties [14]. WJMSCs, harvested from the umbilical cord matrix, have been shown to promote bone regeneration, enhance osteogenic differentiation, and modulate cytokine levels, making them a potential therapeutic option for both bone and cartilage repair. WJMSCs, are known for their low immunogenicity due to their lack of expression of MHC class II molecules and co-stimulatory molecules such as CD40, CD80, and CD86, which is different than other MSCs [15,16,17]. WJMSCs can be efficiently isolated and cultured, demonstrating a high proliferation rate while maintaining their stemness over multiple in vitro passages. They are characterized by hypoimmunogenicity, nontumorigenic properties, and multipotency, with lower heterogeneity compared to MSCs derived from adult tissues [18,19]. Further, WJMSCs regulate the host immune response by suppressing the activation of diverse immune cells [20]. These distinctive characteristics make allogeneic WJMSCs a reliable and safe option for applications in stem cell therapy and tissue engineering. These properties enable WJMSCs to evade immune recognition and reduce the likelihood of eliciting an immune response in xenogeneic models and a clinical trial [21,22]. Preclinical studies have demonstrated that WJMSCs can improve bone healing, reduce inflammation, and protect joint cartilage from degradation, highlighting their dual potential in addressing both bone loss and cartilage damage associated with OP [23]. Furthermore, the immunomodulatory effect of WJMSCs may help to balance the pro-inflammatory cytokines involved in OP, such as the IL-31 and IL-33 axis [24].

Extracorporeal shockwave therapy (ESWT) is a noninvasive, drug- and cell-free method commonly used to treat musculoskeletal disorders [25]. ESWT has demonstrated potential in promoting bone regeneration, enhancing BMD, and protecting joint cartilage in various preclinical studies [26,27]. ESWT exerts local effects on bone strength and systemic effects on bone turnover by modulating molecular pathways, including BMP2 expression and the TGF-β/SMAD2 pathway [26,28]. In particular, site-specific regions of bone can be treated with ESWT, providing targeted therapeutic opportunities for the management of osteoporosis. Moreover, ESWT treatment can lead to an increase in the systemic concentration of osteogenesis factors and bone turnover markers in serum [29,30]. These results suggest that ESWT has not only local effects on bone strength, bone mineral density (BMD), and bone volume but also systemic effects on osteoporosis. In addition, ESWT has been reported to stimulate tissue regeneration through mechanotransduction [31]. Therefore, ESWT has the potential to be a promising noninvasive therapy for osteoporosis by promoting new bone formation and improving bone strength and density, not only locally but also systemically.

Previous research on OP treatments has limitations. Bisphosphonates, though effective in reducing bone resorption, lack the ability to regenerate bone or repair cartilage comprehensively. WJMSCs, while promising for bone and cartilage repair, involve invasive cell preparation and delivery processes, making them less accessible for widespread clinical application. On the other hand, the long-term effects for ESWT remain unclear, with inconsistent findings on its efficacy for different skeletal sites.

Therefore, this study not only evaluates the comparative efficacy of ESWT, bisphosphonate (zoledronic acid), and WJMSC treatments but also demonstrates the potential systemic effects of the three treatments in an OVX rat model of OP. In addition, the IL-33/IL-31 axis is known to play a significant role in the development of osteoporosis [24]. By focusing on the IL-31/IL-33 axis and its role in OP pathology, we investigated the levels of IL31 and IL33 and the pathological changes associated with OP after treatments in an ovariectomized (OVX) rat model. Furthermore, the study compared the efficacy of ESWT with bisphosphonate (zoledronic acid) and Wharton jelly-derived mesenchymal stem cell (WJMSC) treatments for OP, capable of simultaneously addressing bone loss and cartilage degeneration. This study provides new insights into the relative advantages and mechanisms of these therapies, aiming to bridge the limitations of existing OP treatments.

## 2. Materials and Methods

### 2.1. The Animals

All of the animals were treated humanely according to the protocol for the Care and Use of Laboratory Animals, published by the National Institute of Health. All animals were housed under 23 ± 1 °C and had a 12 h light and dark cycle in the Center for Laboratory Animals. The Center for Laboratory Animals was provided for the veterinarians to take care the animals. This study was subjected to the approval of the Institutional Animal Care and Use Committee (IACUC) at the hospital (Approval No: 2018112102) and the work has been reported in line with ARRIVE guidelines [32]. The sample size was calculated using statistical G^*^Power (version 3.1.9.7.) analysis to show that eight rats in each group would provide sufficient statistical power (0.8) to detect a 10% difference in the experiment [33].

### 2.2. Wharton Jelly-Derived Mesenchymal Stem Cells and Cell Identification

**Figure 1:**
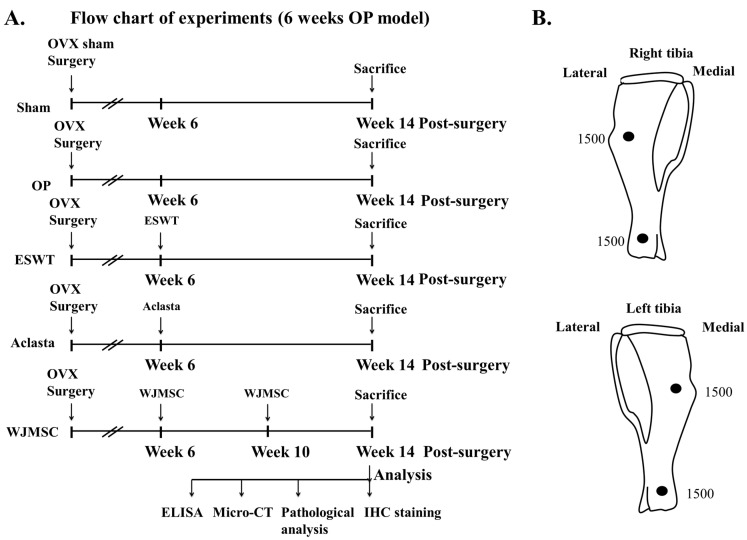
The study design and application of ESWT. (**A**) Flowchart displaying the experiments and timing for knee surgery, shockwave application, Aclasta treatment, WJMSC treatments, and sacrifice of the animals. Eight rats were used for each group. (**B**) Focused ESWT application (0.25 mJ/mm^2^, 4 Hz, 1500 impulses for each location) on right and left tibia at 0.5 cm below the skin of rats at each location, indicated as black circles. OP group = osteoporosis, OVX = ovariectomy, ESWT group = extracorporeal shockwave therapy, WJMSC group = Wharton Jelly-derived mesenchymal stem cell treatment, micro-CT = micro computed tomography, IHC = immunohistochemistry, ELISA = enzyme-linked immunosorbent assay.

Human Wharton jelly-derived mesenchymal stem cells (WJMSC) were purchased from Cellular Engineering Technologies Inc. (Coralville, IA, USA) (Item code: HMSC.WJ-100). The WJMSC were cultured with DMEM complete medium (Gibco, Grand Island, NY, USA) and were identified by surface markers with CD14, CD44, CD105, CD133, and CD166 using a flow cytometer as in our previous study (Supplemental Figure S1) (BD LSRII, Franklin Lakes, NJ, USA) [27]. The dosage and multiple administration of WJMSC cells (2 × 10^6^ cells) were modified as in our previous study, and treatments were administered by tail intravenous injection at 6 and 10 weeks post-surgery (Figure 1A) [34].

### 2.3. Establishment of the Animal Model and ESWT Application

The study design was shown in Figure 1A. In the experiment, forty 10-week old female Sprague-Dawley (SD) rats weighing approximately 200 to 250 g were randomly assigned to each group. The source of shockwaves was a DUOLITH SD1 (Storz Medical AG, Tägerwilen, Switzerland). The shockwaves were applied at two locations of the left tibia and right tibia at 0.5 cm below the skin surface post-surgery at 6 weeks (Figure 1B). Computer-generated randomization was used to assign the animals into 4 groups (8 rats for each group): The Sham group was the sham control without surgery or shockwaves. The OP group received bilateral ovariectomy (OVX), but no ESWT, and served as an osteoporotic animal model. The ESWT group included OVX rats that received a total of 3000 impulses (1500 impulses for each point per tibia) of shockwaves at 0.25 mJ/mm^2^ energy flux density, 4 Hz, on the bone of both tibias as shown in Figure 1B. The Aclasta group included OVX rats that received Aclasta (zoledronic acid, 0.1 mg/kg) by intravenous tail injection. The WJMSC group included OVX rats that received WJMSC cells (2 × 10^6^ cells) by intravenous tail injection at 6 and 10 weeks post-surgery. After surgery, ampicillin (25 mg/kg) and ketorolac (1 mg/kg/day) were administered for five days to prevent infection and relieve pain. All animals were sacrificed at 14 weeks post-surgery (Figure 1A). The evaluating parameters included pathological analysis, micro-CT scanning, and immunohistochemical analysis.

### 2.4. Sample Collection

The rats were sacrificed at 14 weeks post-surgery. The samples were used for pathological and immunohistochemical analysis. Following the disarticulation of the femurs, tibias, knee joints, and spine (T5 to T9), the recovered regions were cleaned and washed with physiologic saline. The gross appearances of the specimens were recorded using a digital camera and the distal section of each specimen was fixed in 4% paraformaldehyde in phosphate buffered saline (PBS) for 24 h. The samples were then decalcified with 20% EDTA (pH 7.4) for 3 weeks at 4 °C and the medium was changed every 3 days. The serum of the rats was prepared after sacrifice at 14 weeks post-surgery and stored at −80 °C until use.

### 2.5. Micro-CT Scan

Cortical and trabecular bone morphometric parameters were determined in the distal femur, tibia, and spine using a micro-CT scan with filter A1 0.5 mm, exposure 270 ms, isotopic pixel size 18 × 18 × 18 μm, X-ray voltage 50 Kv, and 500 μA (SkyScan, 1076, Kartuizersweg 3B 2550 Kontich, Belgium). The bone volume, trabecular thickness, trabecular number, and bone mineral density were measured and computer analyzed.

### 2.6. Histomorphological Examination and Femoral and Tibial Bone Histopathology

The knee and spine samples were subjected to histomorphological examination. The harvested samples were fixed in 4% PBS-buffered formaldehyde at 4 °C for 7 days, and decalcified in 10% PBS-buffered EDTA at 4 °C for 14 days. Decalcified samples were fixed and subjected to paraffin wax embedding and dissection into 5 μm thick sections. The samples were stained with hematoxylin–eosin (HE) and safranin-O. The damage of the cartilage was graded histologically using OARSI scores for the assessments of cartilage structure, cartilage cells, and tidemark integrity. For bone histopathology, femur and tibia bones were fixed in 4% PBS-buffered formaldehyde and stored in decal for 24 h. The femur and tibia bones were sectioned into multiple segments and embedded in paraffin wax blocks. These blocks were subsequently cut using a microtome and stained with hematoxylin and eosin (H&E). After staining, the sections were mounted and examined for histopathological changes.

### 2.7. Immunohistochemistry

Decalcified sections of the knees and spines were probed with primary antibodies against inflammatory-related factors (IL31, 1:50, PAS-114390, Invitrogen, Waltham, MA, USA, and IL33, 1:100, orb 6205, Biorbyt, Cambridge, UK; receptor ST2, 1:50, PA5-23316, Invitrogen, Waltham, MA, USA) and growth factor (BMP2) (1:100, ab6285, Abcam, Cambridge, MA, USA) overnight at 4 °C. Detection was achieved by using a DAB kit (Sigma, Cream Ridge, NJ, USA) followed by counterstaining with hematoxylin. All images of each specimen were captured by using a cool CCD camera (Media Cybernetics, Silver Spring, MD, USA). Images were analyzed by manual counting and confirmed by using an image-pro Plus 6.0 Image-analysis software (Media Cybernetics, Silver Spring, MD, USA).

### 2.8. Enzyme-Linked Immunosorbent Assay

The expression of IL31 (MBS2702983, MyBioSource.com., San Diego, CA, USA), IL33 (M3300, R&D System, Minneapolis, MN, USA), bone morphogenetic protein 2 (BMP2) (DBP200, R&D System, Minneapolis, MN, USA), and vascular endothelial growth factor (VEGF) (RRV00, R&D System, Minneapolis, MN, USA) in the serum of rats was measured by enzyme-linked immunosorbent assay (ELISA) kits.

### 2.9. Statistical Analysis

The statistical analyses were performed using SPSS software (version 17.0, SPSS Inc., Chicago, IL, USA). The data are presented as mean ± standard deviation (SD). Differences between groups were evaluated using one-way analysis of variance (ANOVA) followed by a post-hoc Tukey–Kramer test to determine statistical significance. In order to compare between groups, statistical tests were employed, with significance levels set at *p* < 0.05, *p* < 0.01, and *p* < 0.001.

## 3. Results

### 3.1. ESWT, Aclasta, and WJMSC Modulated the Expression of IL31, IL33, VEGF, and BMP2 in the Serum of OVX Rats

The study design was shown in Figure 1, which involved the application of ESWT on both tibias to stimulate the release of growth factors for bone regeneration, protect the cartilage in joint and spine, and reduce inflammation. We also compared the effects of ESWT, Aclasta (zoledronic acid), and WJMSC treatments on OVX rats.

To assess the efficacy of the treatments, we measured the levels of serum IL31, IL33, VEGF, and BMP2 using ELISA. After treatments, we observed a significant decrease in serum IL31 and IL33 levels, while VEGF and BMP2 levels increased significantly as compared with the OP group (* *p* < 0.05, ** *p* < 0.01, and *** *p* < 0.001) (Table 1). These results indicated that the treatments had differential modulatory effects, with Aclasta demonstrating superior inhibition of IL31 and IL33 expression and greater enhancement of VEGF and BMP2 levels in OVX rats.

### 3.2. Micro-CT Analysis Shows Bone Recovery After ESWT, Aclasta, and WJMSC Treatments in the OVX Rats

**Figure 2:**
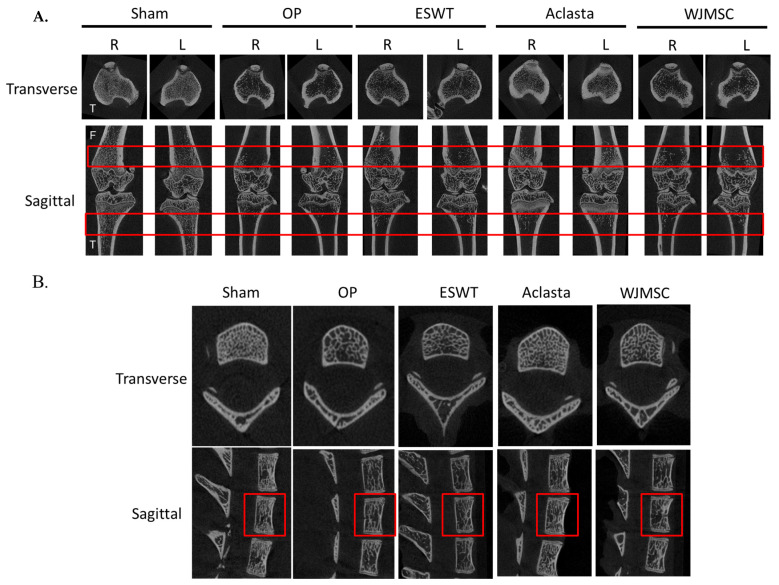
Micro-CT scan of proximal femur, tibia, and spine in different groups. (**A**) The results displayed photomicrographs of the knee in transverse (tibia, up) and sagittal (femur and tibia, below) views from micro-CT. (**B**) The photomicrographs of the spine (T6 to T8) in transverse and sagittal views. The region of the red line was the region of interest in vertebral bone (T7). F indicated the femur and T indicated the tibia. R was the right lower limb and L was the left lower limb. Eight rats were used for each group. OP group was osteoporosis, OVX was ovariectomy, ESWT group was extracorporeal shockwave therapy, and WJMSC group was Wharton jelly-derived mesenchymal stem cell treatment.

In the experiment, micro-CT images were used to visualize the bone structures (lower limbs and spine) of Sham, OP, ESWT, Aclasta, and WJMSC groups (Figure 2). The images of the OP group showed that OVX rats had induced a loss of cancellous bone from the transverse and sagittal views of micro-CT as compared with the Sham group at 6 weeks. After treatments, ESWT, Aclasta, and WJMSC induced bone regeneration in both femurs, tibias, and vertebral bone (Figure 2A,B; red rectangle line, region of interest, ROI). The image results show that ESWT, Aclasta, and WJMSC improved bone regeneration and Aclasta was better than ESWT and WJMSC.

Next, we were further analyzed the ROI regions for bone volume over total volume (BV/TV), trabecular thickness (Tb.Th), trabecular number (Tb.N), and bone mineral density (BMD) (Table 2). In the ESWT, Aclasta, and WJMSC groups, the BV/TV, Tb.Th, Tb.N, and BMD of right and left femurs and tibias as well as spine in the OVX rats were significantly improved as compared with the OP group (* *p* < 0.05, ** *p* < 0.01, and *** *p* < 0.001) (Figure 2 and Table 1). Further, the Aclasta group showed greater improvements in BV/TV, Tb.Th, Tb.N, and BMD compared to the ESWT and WJMSC groups (# *p* < 0.05 and ### *p* < 0.001). The results demonstrated that the treatment administered in Aclasta had better regeneration effects than ESWT and WJMSC in terms of improving the bone recovery of both lower limbs and vertebral bone in OVX rats.

### 3.3. ESWT, Aclasta, and WJMSC Protected the Degeneration of Articular Cartilage, Cancellous Bone, Epiphyseal Plate, Vertebral Cartilage, and Bone in OVX Rats

**Figure 3:**
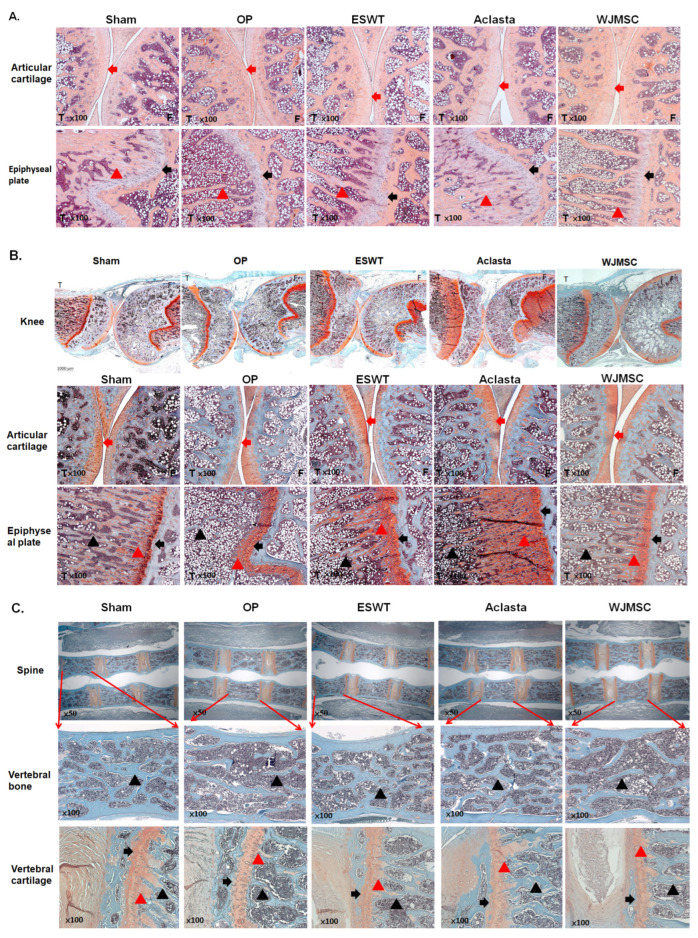
The microphotographs of the left knees and spine. (**A**) The hematoxylin and eosin staining showed the cartilage change in the articular cartilage and epiphyseal plate of the left tibia (×100 magnification) in different groups. (**B**) Safranin-O staining showing the articular cartilage of the left knee (1000 μm) and epiphyseal plate of the left tibia (×100 magnification). (**C**) Safranin-O staining showing the spine (×50 magnification), vertebral bone (×100 magnification), and vertebral cartilage (×100 magnification). Eight rats were used for each group. The red arrowhead indicates cartilage. The black arrowhead indicated epiphyseal plate. The black triangle indicated cancellous bone. The red triangle indicated primary spongiosa. The tibia indicated by T and the femur indicated by F. Osteoporosis indicated by OP group. Extracorporeal shockwave therapy indicated ESWT group and Wharton jelly-derived mesenchymal stem cell treatment indicated WJMSC group.

ESWT, Aclasta (zoledronic acid), and WJMSC treatments were compared the chondroprotective effects on the articular cartilage of the knees in OVX rats. The pathological changes of hyaline cartilage were revealed by H&E stain and safranin-O stain in Sham, OP, ESWT, Aclasta, and WJMSC groups (Figure 3). After treatment, the hyaline cartilages were improved in ESWT, Aclasta, and WJMSC groups as compared with Sham and OP groups (Figure 3B, arrow head). In addition, the epiphyseal plate (black arrow) and region of primary spongiosa (red triangle) became thin and lost the cancellous bone (black triangle) nearby in the OP group (Figure 3B). The epiphyseal plate and cancellous bone were regenerated in ESWT, Aclasta, and WJMSC groups as compared with the OP and Sham groups (Figure 3 and Table 2). The primary spongiosa of the Aclasta group was thicker than in the Sham, ESWT, and WJMSC groups (Figure 3B, red triangle). Furthermore, in the spine of the OP group, there was a loss of vertebral cartilage (arrowhead) and cancellous bone (black triangle). However, following treatments with ESWT, Aclasta, and WJMSC, all of these strictures improved (Figure 3C). Based on our findings, it appears that treatments with ESWT, Aclasta, and WJMSC had a protective effect on various structures including the articular cartilage, cancellous bone, epiphyseal plate, vertebral cartilage, and bone in OVX rats.

### 3.4. ESWT, Aclasta, and WJMSC Modulated the Expression of Bone Remodeling-Related Cytokines in the Articular Cartilage and Epiphyseal Plate of OVX Rats

**Figure 4:**
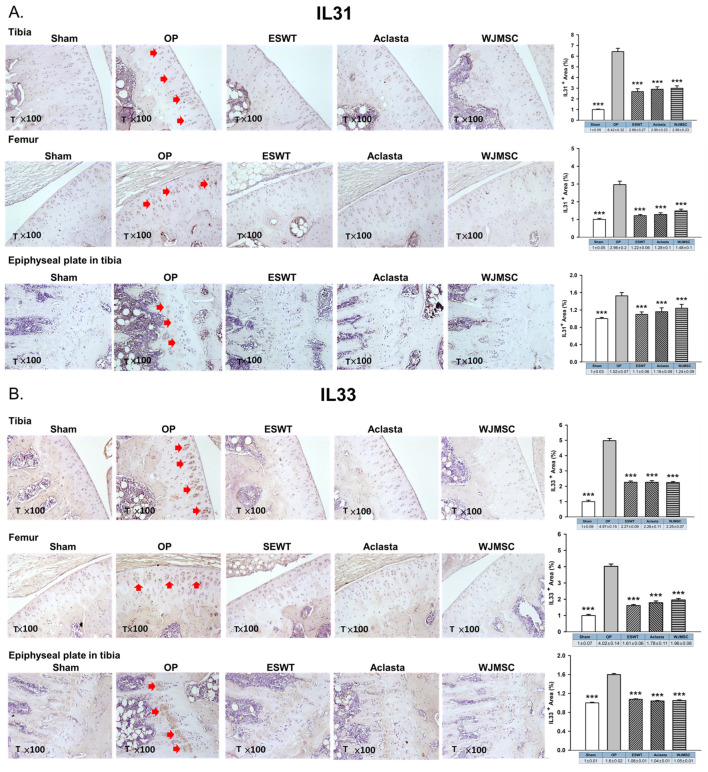

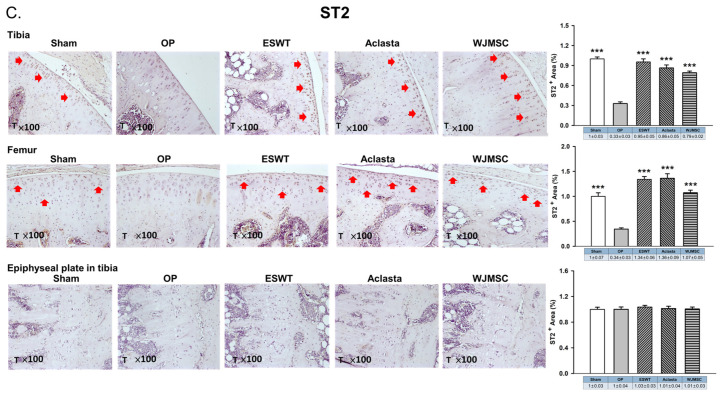
Immunohistochemical analysis for (**A**) IL31, (**B**) IL33, and (**C**) ST2 in the articular cartilage (×100 magnification) of the left knee and epiphyseal plate of the left tibia in Sham, OP, ESWT, Aclasta, and WJMSC groups (right). The expression levels are assessed following the treatments (left). *** *p* < 0.001 as compared with the OP group. Eight rats were used for each group. Osteoporosis indicated OP group. Extracorporeal shockwave therapy indicated ESWT group and Wharton jelly-derived mesenchymal stem cell treatment indicated WJMSC group. T represented tibia. Red arrowhead indicated the expression of proteins.

The cytokines IL31 and IL33 are reported to play a role in bone remodeling and osteoporosis. We showed the expression of IL31, IL33, and IL33 receptor ST2 in the articular cartilage and epiphyseal plate of the left tibia after treatments in OVX rats. The expression of IL31 and IL33 was induced in the articular cartilage and hypertrophic cartilage of the epiphyseal plate in the OP group (red arrow) and reduced in ESWT, Aclasta, and WJMSC groups (*** *p* < 0.001) (Figure 4A,B). There were no significant differences in the expression of IL31 and IL33 among the ESWT, Aclasta, and WJMSC groups. The expression of ST2 in the articular cartilage of the left femur and tibia showed contrasting patterns compared to IL31 and IL33. In the OP group, ST2 expression was reduced, whereas it was significantly induced in ESWT, Aclasta, and WJMSC groups (*** *p* < 0.001) (Figure 4C). The expression of ST2 did not show any significant differences among the ESWT, Aclasta, and WJMSC treatment groups.

### 3.5. ESWT, Aclasta, and WJMSC Stabilised BMP2 Expression to Protect Articular Cartilage, Cancellous Bone, and the Epiphyseal Plate in the OVX Rat Knee

**Figure 5:**
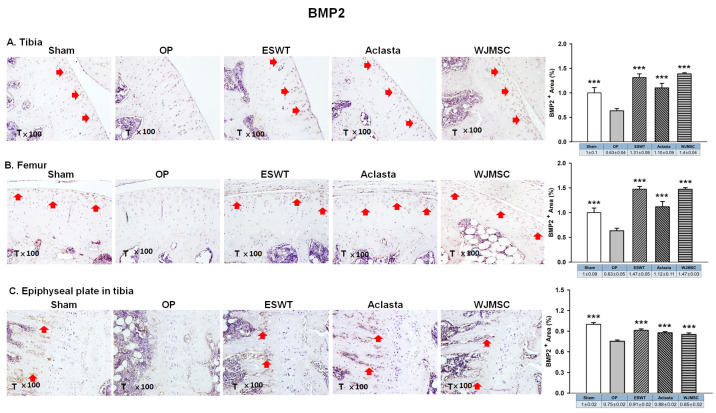
Immunohistochemical analysis for BMP2 in the articular cartilage of the (**A**) tibia, (**B**) femur, and (**C**) epiphyseal plate of the left tibia in Sham, OP, SW, Aclasta, and WJMSC groups (×100 magnification) (right). The expression level was assessed following the treatments (left). *** *p* < 0.001 as compared with the OP group. T represented tibia and Eight rats were used for each group. Extracorporeal shockwave therapy indicated ESWT group and Wharton jelly-derived mesenchymal stem cell treatment indicated WJMSC group. Red arrowhead indicated the expression of proteins.

In the analysis of the left knee, we observed that the expression of BMP2 was reduced in the OP group and was significantly induced to the same level of Sham group following treatments in the articular cartilage of the left tibia and femur (*** *p* < 0.001) (Figure 5). In the epiphyseal plate, the expression of BMP2 was obviously increased in the region of the primary spongiosa of metaphysis in the ESWT, Aclasta, and WJMSC groups to stimulate endochondral bone formation in OVX rats. The results may suggest that ESWT, Aclasta, and WJMSC maintained the level of BMP2 in articular cartilage to protect the extracellular matrix proteins and stimulated the expression in the primary spongiosa of metaphysis to promote the regeneration of endochondral bone and cancellous bone in OVX rats.

### 3.6. ESWT, Aclasta, and WJMSC Modulated the Expression of IL31, IL33, ST2, and BMP2 in the Vertebral Cartilage of OVX Rats

**Figure 6:**
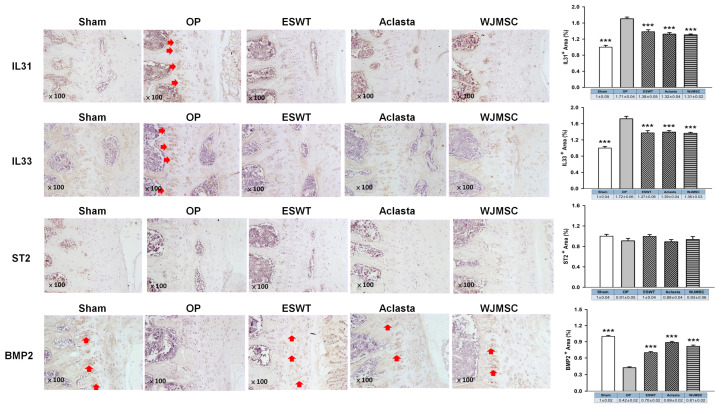
The immunohistochemical analysis of the vertebral cartilage in OVX rats. The immunohistochemical staining (×100 magnification, left panel) and the expression levels (right) of IL31, IL33, ST2, and BMP2 in the vertebral cartilage of the spines for each group. *** *p* < 0.001 as compared with the OP group. Eight rats were used for each group. Extracorporeal shockwave therapy indicated ESWT group and Wharton jelly-derived mesenchymal stem cell treatment indicated WJMSC group. Red arrowhead indicated the expression of proteins.

The expression of IL31 and IL33 was significantly increased in the deep zone of vertebral cartilage in OVX rats, but was decreased in ESWT, Aclasta, and WJMSC groups (*** *p* < 0.001) (Figure 6). However, there was no notable difference in the level of ST2, the receptor for IL33, among all groups. Additionally, BMP2 expression was decreased in the deep zone of vertebral cartilage in OVX rats, but significantly increased in the ESWT, Aclasta, and WJMSC groups to promote endochondral bone formation (*** *p* < 0.001). These findings indicated that while ESWT, Aclasta, and WJMSC groups all led to the inhibition of IL31 and IL33 expression and the enhancement of BMP2 expression, no significant differences were observed between the treatment modalities.

## 4. Discussion

Our study demonstrated that all three treatments, ESWT, Aclasta, and WJMSCs, exerted chondroprotective and osteogenic effects in the ovariectomized (OVX) rat model of osteoporosis. Additionally, the expression of two pro-inflammatory cytokines (IL31 and IL33) was reduced and growth factor (BMP2) was enhanced in the articular cartilage, epiphyseal plate, and spinal cartilage by the widespread effect of the treatments. Interestingly, the levels of serum IL31, IL33, VEGF, and BMP2 were also modulated after treatments, suggesting a potential role for these factors in the pathogenesis and treatments of OP. Overall, these findings provide valuable insights into the potential mechanisms of action of ESWT, Aclasta, and WJMSC in treating OP and may help inform the development of novel therapies for this debilitating disease.

BMP2 and VEGF are significant growth factors for bone formation and bone healing [35,36]. BMP2 is a potent inducer of osteoblastic activity and can stimulate mesenchymal stem cells to differentiate into an osteoblastic lineage, promoting bone formation. Similarly, VEGF has been shown to enhance endochondral bone formation by promoting angiogenesis and the recruitment of chondroclasts to the hypertrophic cartilage, facilitating the replacement of the cartilaginous matrix with a bony callus, ultimately leading to intramembranous ossification [37]. Further, ESWT, Aclasta (zoledronic acid), and WJMSC have been reported to increase the levels of BMP2 and VEGF that are positively associated with bone formation [38,39,40]. Our study findings suggest that all three treatments were effective in stimulating the serum levels of BMP2 and VEGF. This increase in BMP2 and VEGF levels may help to reduce bone loss and promote bone formation throughout the body in OVX rats (Figure 2 and Table 1). Subsequently, all treatments not only prevented bone loss, but also protected the cellular matrix protein in the articular cartilage, epiphyseal plate, and spinal cartilage (Figure 4 and Figure 6). These findings suggest that the treatments may have a beneficial effect on the preservation of bone and joint health. However, further investigations are required to elucidate the mechanisms underlying treatments aimed at promoting bone formation and protecting cartilage.

We know that Aclasta, zoledronic acid, is a type of bisphosphonate drug that is commonly used to treat osteoporosis, especially for postmenopausal women [41]. It works by inhibiting the activity of osteoclasts, the cells responsible for bone resorption, thereby reducing bone loss and improving bone density [42]. In addition, Wharton’s jelly-derived mesenchymal stem cells (WJMSCs) are a type of stem cell that is isolated from the umbilical cord. These stem cells have the ability to differentiate into various cell types, including osteoblasts. When transplanted into a patient, WJMSCs have been shown to improve bone density and promote bone regeneration, making them a potential therapeutic option for osteoporosis [43,44]. Further, ESWT is a well-known and safe physical technology for the bone diseases [26]. Clinical studies have been conducted to investigate the effectiveness of ESWT, zoledronic acid, and WJMSCs for osteoporosis treatment individually [41,43,45]. In addition, zoledronic acid can effectively decrease the incidence of vertebral, hip, and non-vertebral fractures in individuals with osteoporosis. However, treatment with zoledronic acid may also carry the risk of serious side effects such as renal impairment, hypocalcemia, osteonecrosis of the jaw, and atrial fibrillation [46,47]. WJMSCs have been shown to improve bone mineral density and promote bone regeneration in clinical and animal models of osteoporosis; however, the complete side effects remain unknown [23]. Our study demonstrates that ESWT, Aclasta, and WJMSCs show comparable efficacy in promoting systemic bone regeneration in OVX rats, as evidenced by the data presented in Figure 1 and Table 2. The efficacy of Aclasta is better than ESWT and WJMSCs in bone regeneration (Table 2). In terms of overall efficacy, Aclasta appeared to have the greatest impact on systemic bone formation compared to ESWT and WJMSCs. However, all treatments are comparably effective in preventing bone loss across the skeleton. However, considering their comparable efficacy, it may be worthwhile to investigate the potential benefits of combining these treatments to achieve greater bone regeneration and to reduce side effects. ESWT combined with low dose Aclasta might be suggested for osteoporosis. Therefore, further research is necessary to evaluate the safety and efficacy of this combination therapy in human subjects.

Interleukin-33 (IL-33) and interleukin-31 (IL-31) are two cytokines that have been implicated in the pathogenesis of several bone-related disorders, including osteoporosis [24]. IL-33 is an alarmin cytokine, a member of the IL-1 family, and is produced by various cell types such as endothelial cells, fibroblasts, and osteoblasts. IL-33 may play a double-edged role in the regulation of bone remodeling by inducing the differentiation and activation of osteoclasts and osteoblasts [24,48]. IL-33 can stimulate the activity of immune cells that produce inflammatory cytokines, such as tumor necrosis factor-alpha (TNF-alpha) and interleukin-6 (IL-6) [49]. These cytokines can contribute to bone loss by increasing the activity of osteoclasts and suppressing the activity of osteoblasts [50]. On the other hand, IL-33 has been shown to enhance bone formation and prevent bone loss by modulating the expression of IL31, suggesting that it has a protective effect against osteoporosis in animal studies [24]. In addition, IL-31 is a cytokine produced by T-helper 2 (Th2) cells and has been implicated in the pathogenesis of several inflammatory, allergic, and autoimmune disorders [51]. Recent studies have suggested that IL-31 may play a role in bone metabolism by promoting osteoclastogenesis and bone resorption [24]. Studies have shown that IL33 and IL-31 can promote cartilage destruction by inducing the production of matrix metalloproteinases (MMPs) and inhibiting the production of tissue inhibitors of metalloproteinases (TIMPs) in chondrocytes [24,52]. In the experiments, the expression of inflammatory IL31 and IL33 is observed in the articular cartilage of the knee as well as in the hypertrophic zone of the growth plate and spine of OXV rats (Figure 4 and Figure 6). The serum IL-31 and IL-33 levels are increased in OVX rats compared to sham rats (Table 1). These results indicated that osteoporosis-induced IL31 and IL33 systemically disrupted the balance of cartilage homeostasis and endochondral bone formation. In addition, ESWT, Aclasta, and WJMSCs treatments equally reduced the expression of IL31 and IL33 to protect the loss of cartilage matrix in the joints and intervertebral disc. Cartilage protection was an important endpoint in this study, given the dual focus on osteoporosis and its effects on the articular cartilage, epiphyseal plate, and spinal cartilage. Histological analysis demonstrated that all treatments preserved the integrity of the cartilage matrix proteins, preventing the loss of collagen and proteoglycans (Figure 3). This suggests that the treatments not only reduce bone loss but also maintain cartilage homeostasis, which is critical in osteoporotic joints and intervertebral discs.

The limitations of the study are illustrated as follows. The OVX rat model is a commonly used animal model for studying osteoporosis; however, it does not fully elucidate the complexity and symptoms of human osteoporosis. Small animal experiments cannot fully represent the treatment of the human disease. The optimization of ESWT dosage should be further studied and refined in human trials, as the response to the treatments may differ between animals and humans. Moreover, the dosage of WJMSCs requires further optimization in large animal models or human clinical trials. Previous studies have demonstrated that a high dose of WJMSCs (10 × 10⁶ cells/kg, nearly double the dose used in our study) is safe for OVX rats [53]. However, the long-term safety of WJMSC treatment remains to be thoroughly evaluated in future investigations. Further demonstration of the effects of all three treatments is necessary to evaluate their efficacy in treating osteoporosis. Additionally, a more comprehensive analysis of cytokines and growth factors is needed to fully assess the therapeutic potential of these treatments in future clinical trials.

## 5. Conclusions

This study demonstrates that ESWT, Aclasta, and WJMSC treatments improve bone regeneration and prevent the degeneration of articular and vertebral cartilage in osteoporotic rats. The three therapies reduced IL31 and IL33 expression in articular and vertebral cartilage while inducing the IL33 receptor ST2 in articular cartilage. The levels of IL31 and IL33 in serum were decreased, while BMP2 and VEGF were increased, enhancing bone formation in limb and vertebral bones. BMP2 modulation in cartilage further supports their role in osteogenesis. In addition to the need for effective osteoporosis treatments with fewer side effects, our findings suggest that combining ESWT with lower doses of Aclasta or WJMSC may enhance bone regeneration while minimizing risks like renal impairment or osteonecrosis. However, the study has limitations. It is restricted to a preclinical OVX rat model, with unknown applicability to humans. The molecular pathways underlying cytokine modulation remain unclear, and the long-term safety of combination therapies needs validation. Future studies should confirm these results in large animal models or clinical trials, investigate the mechanisms of cytokine and growth factor regulation, and optimize dosing regimens to enhance safety and efficacy. Addressing these challenges will lead to the development of safer and more effective osteoporosis treatments for clinical use.

## Figures and Tables

**Table 1:** The serum levels of IL31, IL33, VEGF, and BMP2 in the OVX rat from Sham, OP, ESWT, Aclasta, and WJMSC groups by ELISA at 14 weeks post-surgery.

	Groups	Sham	OP	ESWT	Aclasta	WJMSC
Biomarkers						
IL31 (pg/mL)		87 ± 1.2 **	102 ± 2.2	85 ± 3.6 **	97 ± 0.9 #	92 ± 3.4 *
IL33 (pg/mL)		48 ± 3.4 *	56 ± 0.9	51 ± 1.7 *	51 ± 1.7 *	56 ± 1.7 #
VEGF (pg/mL)		279 ± 12.3 ***	232 ± 2.4	247 ± 5.6 *	263 ± 4.8 ***/#	271 ± 3.6 ***/#
BMP2 (pg/mL)		102 ± 6.4 *	73 ± 6.9	91 ± 3.6 *	105 ± 6.5 **	111 ± 8.6 **

The results are given as mean ± SD. Eight rats were used for each group. * *p* < 0.05, ** *p* < 0.01, and *** *p* < 0.001 as compared with the OP group. Extracorporeal shockwave therapy is indicated with ESWT and Wharton jelly-derived mesenchymal stem cell is indicated with WJMSC. # *p* < 0.05 as compared with ESWT for Aclasta and WJMSC.

**Table 2:** The micro-CT analysis of both lower limbs and spine in Sham, OP, ESWT, Aclasta, and WJMSC groups.

Micro-CT Data	Tibia				
Limbs	Left				
Groups	Sham	OP	ESWT	Aclasta	WJMSC
BV/TV (%)	50.722 ± 1.316 ***	21.988 ± 1.573	26.764 ± 1.095 *	37.227 ± 0.566 ***#	27.501 ± 1.349 *
Trabecular thickness (mm)	0.0841 ± 0.002 **	0.0963 ± 0.0029	0.0886 ± 0.0009 *	0.0849 ± 0.0012 **	0.0903 ± 0.0017
Trabecular number (mm)	5.903 ± 0.226 ***	2.312 ± 0.199	2.957 ± 0.094 *	4.522 ± 0.107 ***#	3.013 ± 0.168 *
BMD (g/cm^3^)	0.4933 ± 0.024 ***	0.0048 ± 0.011	0.0847 ± 0.012 **	0.226 ± 0.014 ***###	0.082 ± 0.014 **
Limbs	Right				
Groups	Sham	OP	ESWT	Aclasta	WJMSC
BV/TV (%)	50.506 ± 1.452 ***	20.965 ± 1.449	28.281 ± 1.091 *	37.482 ± 0.801 ***#	27.885 ± 1.267 *
Trabecular thickness (mm)	0.0876 ± 0.0013 **	0.0989 ± 0.0027	0.0912 ± 0.0007 *	0.0847 ± 0.0009 ***	0.0928 ± 0.0013
Trabecular number (mm)	5.683 ± 0.189 ***	2.138 ± 0.137	3.085 ± 0.110 *	4.399 ± 0.097 ***#	2.947 ± 0.151 *
BMD (g/cm^3^)	0.465 ± 0.027 ***	0.023 ± 0.018	0.098 ± 0.010 *	0.236 ± 0.016 ***###	0.091 ± 0.016 *
**Micro-CT data**	**Femur**				
Limbs	Left				
Groups	Sham	OP	ESWT	Aclasta	WJMSC
BV/TV (%)	50.705 ± 0.617 ***	28.153 ± 2.650	31.867 ± 0.826	41.085 ± 0.931 **#	32.599 ± 0.856
Trabecular thickness (mm)	0.095 ± 0.002 *	0.108 ± 0.004	0.099 ± 0.001	0.108 ± 0.001	0.104 ± 0.002
Trabecular number (mm)	5.449 ± 0.103 ***	2.586 ± 0.209	3.141 ± 0.053 *	3.947 ± 0.083 ***	3.328 ± 0.079 *
BMD (g/cm^3^)	0.595 ± 0.025 ***	0.089 ± 0.025	0.175 ± 0.014 *	0.337 ± 0.007 ***#	0.196 ± 0.016 *
Limbs	Right				
Groups	Sham	OP	ESWT	Aclasta	WJMSC
BV/TV (%)	48.958 ± 0.684 ***	30.426 ± 3.172	33.241 ± 0.718	42.533 ± 0.432 *#	34.240 ± 1.049
Trabecular thickness (mm)	0.092 ± 0.002	0.115 ± 0.004	0.101 ± 0.001	0.107 ± 0.000	0.105 ± 0.002
Trabecular number (mm)	5.413 ± 0.125 ***	2.632 ± 0.222	3.334 ± 0.028 *	4.023 ± 0.061 ***#	3.191 ± 0.079 *
BMD (g/cm^3^)	0.578 ± 0.023 ***	0.094 ± 0.021	0.183 ± 0.011 *	0.352 ± 0.008 **#	0.204 ± 0.015 *
**Micro-CT data**	**Spine (T7)**				
Groups	Sham	OP	ESWT	Aclasta	WJMSC
BV/TV (%)	49.425 ± 1.447 **	37.188 ± 1.384	40.869 ± 1.555 *	43.727 ± 1.104 *#	37.039 ± 1.693
Trabecular thickness (mm)	0.110 ± 0.003	0.107 ± 0.002	0.109 ± 0.003 *	0.110 ± 0.003 *	0.110 ± 0.003 *
Trabecular number (mm)	4.499 ± 0.044 **	3.476 ± 0.125	3.731 ± 0.149 *	3.976 ± 0.138 *	3.364 ± 0.102
BMD (g/cm^3^)	0.314 ± 0.012 ***	0.195 ± 0.011	0.253 ± 0.014 ***	0.266 ± 0.011 ***	0.227 ± 0.014 ***

The results are given as mean ± SD. L = left tibia and R = right tibia. Eight rats were used for each group. * *p* < 0.05, ** *p* < 0.01, and *** *p* < 0.001 as compared with the OP group. # *p* < 0.05 and ### *p* < 0.001 as compared with ESWT for Aclasta and WJMSC groups.

## Data Availability

The datasets used and/or analyzed during the current study are available from the corresponding author on reasonable request.

## Supplementary Materials

The following supporting information can be downloaded at: https://www.mdpi.com/article/10.3390/biomedicines12122823/s1, Supplemental Figure S1. The Human WJMSC surface markers are measured by flow cytometry. The positive markers are CD44 (99.71%), CD105 (99.66%) and CD166 (99.97%). The negative markers are CD14 and CD133.
