# Supplementary material for: Comparative Analysis of Extracorporeal Shockwave Therapy, Bisphosphonate, and Wharton Jelly-Derived Mesenchymal Stem Cells in Preserving Bone and Cartilage Integrity and Modulating IL31, IL33, and BMP2 in the Cartilage of Ovariectomized Rat Model
Source: Biomedicines. 2024 Dec 12;12(12):2823. doi: 10.3390/biomedicines12122823 (PMC11673226; doi:10.3390/biomedicines12122823)
Supplement: Supplementary file 1 [file biomedicines-12-02823-s001.zip › biomedicines-3331760-supplementary.pdf]

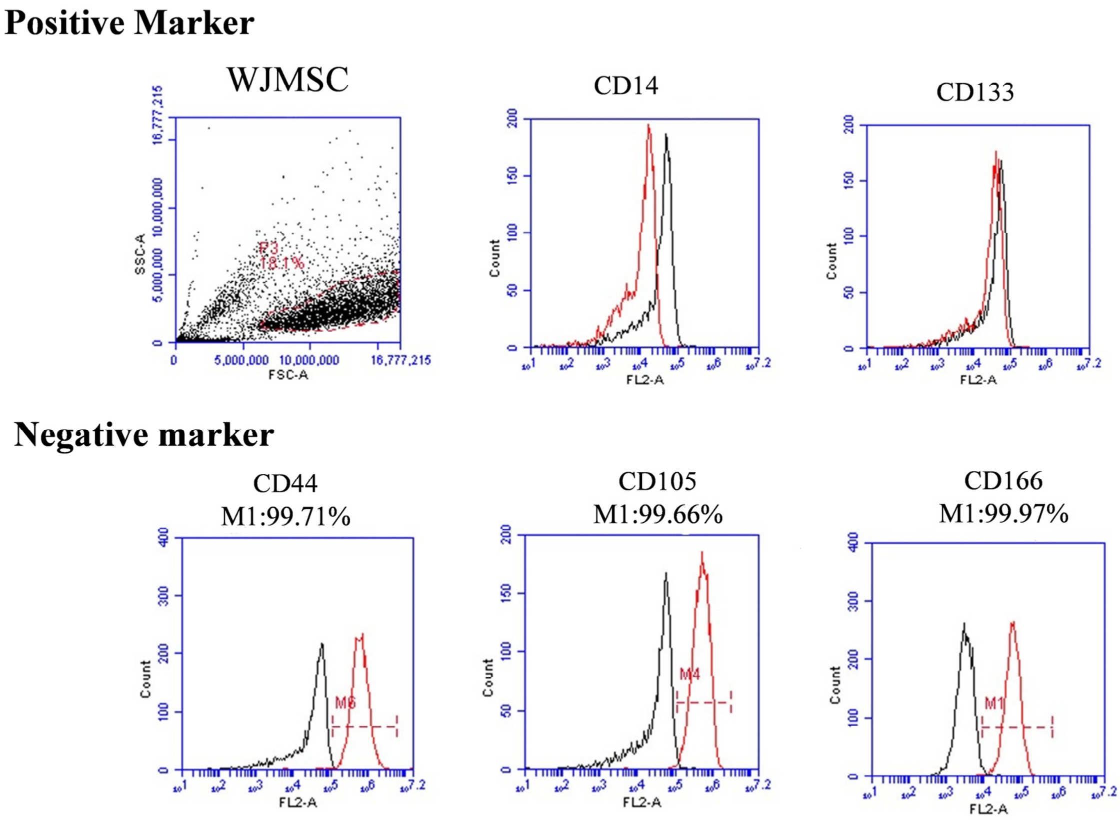


**Supplemental Figure S1.** The Human WJMSC surface markers are measured by flow cytometry. The positive markers are CD44 (99.71%), CD105 (99.66%) and CD166 (99.97%). The negative markers are CD14 and CD133.
